# Correction: Iwahashi et al. Pathophysiological Roles of the CX3CL1-CX3CR1 Axis in Renal Disease, Cardiovascular Disease, and Cancer. *Int. J. Mol. Sci.* 2025, *26*, 5352

**DOI:** 10.3390/ijms27093945

**Published:** 2026-04-29

**Authors:** Yuya Iwahashi, Yuko Ishida, Naofumi Mukaida, Toshikazu Kondo

**Affiliations:** 1 Department of Forensic Medicine, Wakayama Medical University, 811-1 Kimiidera, Wakayama 641-8509, Japan; 0911yuya@wakayama-med.ac.jp (Y.I.); mnao.otcyt@gmail.com (N.M.); 2 Department of Urology, Wakayama Medical University, Wakayama 641-8509, Japan

In the original publication [1], Ref. 36 “Alajoleen, R.M.; Oakland, D.N.; Estaleen, R.; Shakeri, A.; Lu, R.; Appiah, M.; Sun, S.; Neumann, J.; Kawauchi, S.; Cecere, T.E.; et al. Tlr5 Deficiency Exacerbates Lupus-Like Disease in the Mrl/Lpr Mouse Model. *Front. Immunol.* **2024**, *15*, 1359534” was included unnecessarily and has now been removed. Consequently, all subsequent references have been renumbered, and the in-text citation numbers have been updated accordingly. The citation has now been deleted in 2.2. Glomerulonephritis, Paragraph 3 and should read:

Investigations on glomerulonephritis have also been carried out in animals; MRL/lpr mice spontaneously develop glomerulonephritis with increased circulating immune complexes, autoantibody production, and cytokine abnormalities [35]. The glomerular lesions seen in MRL/lpr mice are diffuse, cell-proliferative and/or wire loop-like lesions and are a frequently used animal model, as they resemble the various histological patterns observed in human lupus nephritis [36,37]. CX3CL1 expression and CD16+ monocyte accumulation in glomeruli were associated with histopathological features of glomerular lesions in MRL/lpr mice [38]. In addition, lupus nephritis showed functional and histological improvement after treatment with anti-CX3CL1, indicating a therapeutic effect of anti-CX3CL1 [38].

The authors state that the scientific conclusions are unaffected. This correction was approved by the Academic Editor. The original publication has also been updated.
